# Determinants of reproductive health services utilization among rural female adolescents in Asgede-Tsimbla district Northern Ethiopia: a community based cross-sectional study

**DOI:** 10.1186/s12978-019-0664-2

**Published:** 2019-01-11

**Authors:** Hailay Gebreyesus, Mebrahtu Teweldemedhin, Abebe Mamo

**Affiliations:** 1 Department of Public Health, College of Health Science, Aksum University, P.O. Box 298, Aksum, Ethiopia; 2 Department of Medical Laboratory Sciences, College of Health Science, Aksum University, Aksum, Ethiopia; 3 Departments of Health Education and Behavioral Sciences, Jimma University, Jimma, Ethiopia

**Keywords:** Determinants of female adolescent, Reproductive health services, Utilization

## Abstract

**Background:**

Adolescents especially females in rural area are vulnerable to a wide range of reproductive health problems including sexually transmitted infections, unwanted pregnancies, and unsafe abortion. They have limited access to reproductive health services that focus on the special needs of female adolescents. This study was aimed to assess the determinants of reproductive health service utilization among rural female adolescents of Asgede-Tsimbla district.

**Methods:**

A community-based cross-sectional study was carried out from February to April 2018, in eight randomly selected sub-districts of Asgede-Tsimbla. A total of 844 female adolescents aged 15–19 were interviewed using a pre-tested structured questionnaire. Data were entered into Epi-info Version 3.5.3 and then exported to SPSS Version 21 for analysis. Bivariate and multivariate logistic regression analysis was carried out to assess the association. Statistical significance was declared by 95% confidence interval of the odds ratio.

**Result:**

From 844 participants, 95.5% of female adolescents heard about reproductive services from different sources and 69.7% of them utilized the reproductive health services within the last 12 months. Factors like, age of 16–20 years (AOR = 1.85, 95%CI: 1.17–2.92), mother’s educational status (being illiterate (AOR = .33, 95%CI:.14–.77)), discussion about reproductive health services with their family (AOR = 8.02, 9%CI:5.52–11.66), being Merchant (AOR = 2.7995%CI:1.11–6.96), unemployed (AOR = 2.90, 95%CI:1.19–7.06) or student (AOR:2.38, 95%CI:1.04–5.42) in occupation, high perceived severity (AOR = 4.05, 95%CI:2.68–6.11), high perceived barriers (AOR = .44, 95%CI:.30–64) were independent predictors of reproductive health services utilization among female adolescents the study area.

**Conclusion:**

About 69.7% of the adolescent females were utilizing reproductive health services in the study area though it was very low as compared with the national plan. Introducing messages that increase the perceived threat and decreasing perceived barriers to utilize reproductive health services as well as increasing self-efficacy of adolescent females would help further increase reproductive health services utilization by adolescent females.

## Plain English summary

Even though the government of Ethiopia has been putting tremendous efforts to promote the adolescent and youth-friendly reproductive health services, few national and regional studies showed that utilization of female adolescent reproductive health services is still very low. So, this study was aimed to find out the determinants of reproductive health services utilization among female adolescents in Asgede-Tsimbla district of Northern Ethiopia.

Female adolescents utilized as well as utilizing reproductive health service before the past 12 months and during the actual data collection were included in the study. Among 27 Smallest Administrative areas of the district, eight were randomly selected, and a total of 844 participants were needed. Reproductive health services Utilization was measured if female adolescents utilized one of the three given components (FP, abortion care and VCT). Data were entered into Epi-info Version 3.5.3 and exported to SPSS version 21 for analysis. Adjusted odds ratios with the 95% confidence interval and a *p*-value of less than 0.05 were considered to decide significant association between the outcome and the independent variables. In this study, only 69.7% of female adolescents utilized PH services within the last 12 months. Living arrangement, discussion with their family about RH services and family marital status, were independent predictors of RH services utilization among female adolescents in the study area. The overall utilization of RH services was very low in the study area as compared with the national plan. There should be community-based health promotion activities in order to reach every segment of the adolescent population.

## Background

Adolescence is an individual’s aged from 10 to 19 years. It is a period of transition from childhood to adulthood and characterized by significant physical, psychological, and social behavioral changes that may place their life at high risk [1, 2]. Globally, about 45% of all new human immunodeficiency virus (HIV) infections occurred among people aged 15–24 years. In Africa alone, an estimated 1.7 million young people were exposed to many reproductive health problems [3], and 60% of all new HIV infections occur in adolescents who are15–19 years old [4, 5].

About 85% of the world’s young populations aged 10–24 years are living in developing countries. In Ethiopia, they account for 35% of the total population [6]. Female adolescents, particularly in the rural parts of the developing countries, are vulnerable to a wide range of reproductive health problems (RHP) such as sexually transmitted infections; unwanted and early pregnancies and unsafe abortion due to an early and unprotected sexual activity and misconceptions on sexual risk behaviors. About 54% of young girls in Ethiopia had experienced sexual violence and commercial sex work had become common phenomena among young girls [7–9]. According to world health organization report shows that about 54% in Ethiopia and 48% in Bangladesh female adolescents aged 15–19 years had experienced sexual violence within the last 12 months respectively [7].

For instance, in sub-Saharan Africa including Ethiopia, more than half of girls aged 15–19 years were sexually active and more than half of them gave birth before the age of 20. Each year, about 15 million adolescents aged 15–19 years gave birth and a large proportion of these pregnancies were unplanned, as many as 4 million faced unsafe abortion, and up to 100 million were affected by sexually transmitted disease [10–13].

Moreover, around 70,000 female adolescents with early-marriage die each year due to pregnancy and childbirth-related complications [11]. The risks of dying from complications related to pregnancy or childbirth are two times higher for those aged 15–19 and five times higher for those aged 10–14 than for women in the mid-twenties [14].

Ethiopia is one of the nations that have the highest early marriage practice. In 2010, the number of adolescent girls who were in the age range of 10–19 years had been exposed for forced marriage constituting 49.2% of the target population [15]. Forty-five percent of the total birth in Ethiopia occurs among adolescent girls and young women [16]. Unsafe abortion is a leading cause of maternal death and disabilities and 54% of pregnancies in female adolescents under the age of 15 years were unwanted; they are three times more likely to end their pregnancy with abortion [17–19]. Another study conducted in Ethiopia shows that 24.4% female adolescent had an unwanted pregnancy and 89% of them had the history of abortion [20].

Even though Female adolescents in developing countries are at increased risk for negative reproductive health outcomes [2, 4, 6]; they did not use reproductive health services as compared with women aged greater than 20 years [21–23]. Among married female adolescents who are 15–19 years old, only 17% practice family planning methods, and the use of contraception is believed to even lower among unmarried sexually active female adolescents [22].

According to the 2016 Ethiopian Demographic and Health Survey report, 13% of the adolescent females aged 15–19 years have begun childbearing and a majority of them are living in rural areas [20]. Overall, 45% of the sexually active unmarried women are currently not using any family planning methods. Among sexually active adolescents who aged 15–19 years, women and men who were tested for HIV were only 22 and 18% respectively [20]. Another study conducted in Ethiopia among female adolescents indicated that 32.9% of the sexually active unmarried young females use a condom [20].

Considering the risks of RH problems, the government of Ethiopia has introduced and currently implementing a policy that provides free reproductive health services to all adolescents in all governmental healthcare facilities. However, different studies conducted in the country revealed that reproductive health services utilization among female adolescents is still very low as compared to the national health policy [21–23].

There was limited information regarding the levels of reproductive health service utilization and its determinants among female adolescents, particularly in the study area. Thus, this study was aimed to find out the determinants of reproductive health services utilization within this age category, especially family planning, safe abortion care, and Voluntary counseling and testing service for HIV using Health Belief Model.

### Health belief model (HBM)

The present study applied the HBM perspective to determining RH services utilization in a sample of Ethiopian rural adolescent girls of Asgede-Tsimbla district. The underlying concept of the HBM is that health behavior is determined by personal beliefs or perceptions about a problem and the strategies available to decrease its occurrence. Personal perception is influenced by the whole range of interpersonal factors (such as mental disorders, substance abuse, and domestic violence) affecting health behavior [24].

Cognitive theorists, however, emphasize the role of subjective hypotheses and expectations held by individuals, believing that behavior is a function of the subjective evaluation of the problem and of the subjective probability, that a particular action will achieve a positive outcome, which could be generated from the idea that mental processes such as thinking, reasoning and hypothesizing are critical components of all cognitive theories [25].

Therefore, utilization of health services was further delineated in terms of the individual’s estimates of personal susceptibility to and perceived severity of RH problems and evaluating the cost-benefit analysis wherein individuals weigh the action’s expected benefits with perceived barriers, as well as the likelihood of being able to reduce that threat through personal action or self-efficacy. In addition, there are modifying factors that can affect rural female adolescents in utilizing RH services and these factors would include knowledge, sources of information, personal relationships, incentives, and living arrangement [26].

Therefore, the aims of used Health Belief Model (HBM) in this study was to understand determinants of RH services utilization in a sample of Ethiopian rural adolescent girls of Asgede-Tsimbla district and possible reasons for none or underutilization.

## Methods

### Study design and setup

A community-based cross-sectional design was adopted in this study, to find out the determinants of reproductive health services utilization among female adolescents in Asgede-Tsimbla district, Northern Ethiopia. Asgede-Tsimbla district is found North West Zone of Tigray Regional State, 1107 km of Addis Ababa city. According to 2018 official report, the district has a total population of 180,161(male 91,663 and 88,498 female), and adolescents 15–19 years were estimated to be 15% (27,024) of which 93% them are living in the rural area. The district has 27 sub-districts (Smallest Administrative Unit), one primary hospital, six health centers and, 25 health posts. Asgede-Tsimbla was selected by the Ministry of Agriculture and Rural Development in 2004 as an area for voluntary resettlement for farmers from overpopulated areas. In addition, there are nine identified gold mining areas and refugees’ camps. To use this, advantage, there are large numbers of heterogeneous populations from those about 92% are rural dwellers including adolescent females come from different parts of the Tigray region including nearby zones.

The participants of this study were unmarried female adolescents 15–19 years old of the selected kebeles of the district, who lived in the area for a minimum of 6 months including in the study. Those female adolescents’ age 15–19 years who are unable to communicate due to their physical and mental illness were excluded from the study. The study was conducted from February to April 2018.

### Sample size and sampling procedure

The sample size was calculated using a formula for estimation of a single proportion as follow:

$$ n=\frac{{\left( Za/2\right)}^2\mathrm{p}\left(1-p\right)}{d^2} $$

Where

- n = the final sample size of the study
- Z = Standard normal variable at 95% confident level (1.96)
- d = Marginal of error (0.05)
- p = Expected prevalence of females adolescent who was utilized sexual reproductive health services (50%) and a design effect of 2. Then 10% was added for the expected non-response, making the final sample size 844.

From the 27 Smallest Administrative areas (sub-districts) of Asgede-Tsimbla district, eight were randomly selected. The sample allocation was proportional to the household size of each sub-district. The first household from each sub-district was identified using the lottery method, and then a systematic random sampling technique was applied to identify the next households to be included. Female adolescents who were found in the selected households were interviewed. In the case of more than one eligible participant in the household, the lottery method was used to select one. In households in which adolescents were not at home, but it was known that there were eligible adolescents for the study, the interviewers revisited the household at three different time intervals, and when interviewers failed to meet the adolescent, the household was excluded from the survey and replaced by the next household in a clockwise direction.

### Data collection tools and procedure

An anonymous, structured, interview questionnaire was developed after reviewing relevant literatures that reviewed key variables as well as earlier studies on sexual reproductive health (SRH) problems among adolescents [1, 3, 19–28]. Some questions were also adapted from Integrated Family Health (IFH) guidelines for youth and modified to our local area [29, 30].

The questionnaire and consent document was first developing in English, then translated into Tigrigna (the local language), and finally retranslated into English by another translator to check consistency. Before the actual work, eight data collectors who were female health extension workers and diploma graduated from nursing whose age not more than 24 years. There were also two supervisors who were masters in public health (MPH) to support, follow and check the activities of data collectors. Two days intensive training was given to data collectors and supervisors on the research ethics, the study objectives, data collection principles, and processes, as well as the research instruments. Five percent of the questionnaire was pre-tested for clarity and ascertain internal consistency after selecting one rural kebele which was not included in the actual study. After analyzing the pre-test result necessary modifications were made accordingly before using it in the actual survey. The interviewers were the same gender of the actual respondents in order to minimize embarrassment as some of the questions were about personal sexual lifestyle issues.

The Authors coordinated the data collection, made site visits, and oversaw the whole process and then if any inconsistency and errors were checked and solved immediately.

The questionnaire was grouped into four categories; socio-demographic characteristics (16 items); RHS utilization (5 items); knowledge about RHS (5 items); attitude about RHS (2 items) and perceptions about RHS using Health Believe Module (HBM) constructs; including perceived susceptibility (10 items), perceived severity (6 items), perceived barrier (10 items), perceived benefit (10 items), cue to action (4 item), self efficacy (10 item) were assessed in this study.

All knowledge questions were summed up and treated as a continuous variable and those participants with high score considered as more knowledgeable. The reliability coefficient of knowledge in utilizing reproductive health services was 0.66.

For HBM constructs except for cues to action all items were assessed with five-point Likert-scale response in which the choices ranging from “strongly disagree (1 point)” to “strongly agree (5 points)” and all constructs were treated as continuous variables. The reliability coefficient of the constructs of Health Belief Model for this study were 0.81, 0.78, 0.76 and 0.64 for perceived susceptibility, seriousness, barriers and benefits to a utilization of reproductive health services respectively.

Utilization of reproductive health services also treated as those female adolescents who received at least one component of RH services by health care providers at any governmental or private institutions within the last 12 months. This was measured through the dichotomous response (yes or no) by asking whether participants had utilized one or more sexual reproductive health services components within the last 12 months. If the response of participant was “yes” further validated with questions on the types of SRH services utilized. Reproductive health services, particularly in this study, were safe abortion care, FP and VCT. Abortion care service utilization was defined as any termination of pregnancy under the supervision of skilled attendant including midwifery, nurse, and medical doctor. FP service utilization was defined as ever use of any modern contraceptives within the last 12 months, and VCT service utilization referred to ever utilization of VCT service for HIV testing. Finally, a positive (“yes”) response to any one of these services was observed as service utilization.

Perceived susceptibility: an individual’s assessment of his or her chances of getting the disease or problem.

Perceived severity or seriousness: an individual’s judgment that the disease was sever.

Perceived benefits: an individual’s conclusion as to whether the new behavior was better than that he or she already doing.

Perceived barrier: An individual’s opinion that would stop him or her from adopting the new behavior.

Self-efficacy: Beliefs about one’s ability to perform the recommended response.

Cue to action: Those factors that would start a person on the way to change behavior or strategies to activate one’s readiness to utilize any services.

### Data processing and analysis procedures

The data gathered through the pre-tested structured questionnaire was checked and coded manually for completeness. Questionnaires which were incomplete for key variable/s have been excluded from the study. The coded data was entered in double, checked for missing value and outliers (Epi-info Version 3.5.3) and then exported to SPSS Version 21 for analysis. Frequencies mean and percentage were used to describe the data. Binary logistic regression was carried out. Crude association between dependent and independent variables was assessed by bivariate logistic regression and its strength was presented using odds ratio and 95% confidence intervals. Bivariate followed by multivariate logistic regression analysis was also carried out to control confounding effects of variables. Variables with a *p*-value less than 0.25 in binary logistic regression were entered into multiple logistic regressions. In multivariate analysis, variables having *p*-value less than 0.05 were considered as significantly associated with the outcome variables. Adjusted odds ratio (AOR) with 95% CI was used determine the presence and strength of association.

## Result

### Socio-demographic characteristics

Out of the 844 girls, a total of 841 (99.6%) female adolescents participated in the study. Their mean age was 17.35 ± 1.63 years. Majority of the female adolescent 727 (86.1%) were living with both their father and mother and 639 (75.7%) of the respondent’s parents were farmer (Table 1).

**Table 1:** Distribution of socio-demographic characteristics of female adolescents in Asgede-Tsimbla district, Northern Ethiopia, 2018

Variables	Frequency [*N* = 844]	Percentage [%]
Age		
1. 10–15	188	22.3
2. 16–20	656	77.7
Educational level		
1. Only read and write	41	4.9
2. Grade 1–8	442	52.4
3. Grade 9–12	29	3.4
4. College and above	332	39.3
Religion		
1- Orthodox Christians	739	87.6
2- Muslim	105	12.4
Living arrangement		
1- With Father and Mother	727	86.1
2- With Father or Mother only	65	7.7
3- With relatives/friends	13	1.5
4- Alone	38	4.6
Current occupation		
1- Government Employee	41	4.9
2- Private Employee	73	8.6
3- Merchant	89	10.5
4- Unemployed	166	19.7
5- Student	475	56.3
Father’s Educational Status		
1- Illiterate	77	9.1
2- Read and write	692	82.2
3- Elementary school	49	5.8
4- Secondary School	17	2.0
5- College and above	9	1.1
Mother’s Educational Status		
1- Illiterate	121	14.3
2- Read and write	515	61.0
3- Elementary school	82	9.7
4- Secondary School	75	8.9
5- College and above	51	6.0
Parent’s Occupation		
1- Government employee	37	4.4
2- Merchant	168	19.9
3- Farmer	639	75.7
Marital Status of The Family		
1- Live together	704	83.4
2- Divorced	39	4.6
3- Widowed	101	12.0

### Knowledge

The Knowledge scores were ranging from 8 to 18 with the mean score of 12.56 ± 3.0. Of the 844 participants, 806 (95.5%) female adolescents heard about reproductive health services from different sources, a majority of the respondents 342 (42.4%) heard from radio and followed by teachers 204 (25.3%).

Out of the 844 participants, 588 (69.70%) females utilized reproductive health services within the last 12 months. Concerning the type of service, 455 (77.3%) used family planning, 53 (9%) received health education service about reproductive health services, 45 (7.6%) used STI care, 19 (3.2%) used abortion care, and 16 (2.7%) used VCT services (Table 2).

**Table 2:** Source of information and utilization of reproductive health services among female adolescents in Asgede-Tsimbla district, Northern Ethiopia, 2018

Variables	Frequency [*N* = 844]	Percentage [%]
Heard about reproductive health service		
1- Yes	806	95.5
2- No	38	4.5
Sources of information		
1- Radio	342	42.4
2- Teacher	204	25.3
3- Health worker	53	6.6
4- Family	41	5.1
5- TV	19	2.4
6- From two or more of the above sources	147	18.2
Type of services utilized		
1. Family planning	455	77.4
2. Health education on reproductive health services	53	9.0
3. STI care	45	7.7
4. Abortion care	19	3.2
5. VCT	16	2.7
Preference of service providers by girls		
1. Health Extension Workers	321	39.5
2. Medical Doctors	279	33.1
3. Nurses	153	18.1
4. Religious Leaders	78	9.3
Discussion about reproductive health service within a family		
1. Yes	436	51.7
2. No	408	48.3

### Perception about reproductive health service utilization

The perception of the female adolescents about the reproductive health service utilization was addressed using health belief model constructs and all the constructs were assessed through the Likert scale. The mean score for perceived severity was 20.7 with SD of 11.4. Four hundred thirty-eight (69.1%) of female adolescents had high score of perception towards severity of RH problems. Twenty seven (31.3%), 48 (30.6%), 94 (27.6%) Female adolescents had low score of perceived susceptibility and perceived barrier, for reproductive health problems (Table 3).

**Table 3:** The distribution of mean score of perceptions on reproductive health service utilization among female adolescents, Asgede-Tsimbla District, Northern Ethiopia, 2018

Perceptions	Mini. Value	Max. Value	Mean[±SD]
Susceptibility	13	38	33.1 [8.8]
Severity	6	30	20.7 [11.4]
Barriers	16	34	26.9 [2.9]
Benefits	24	41	39.2 [2.9]
Self-Efficacy	19	41	28.6 [3.9]
Cues To Action	3	5	3.8 [.76]

### Predictors of reproductive health services utilizations

After the Bivariate lodistic regression, Multivariate logistic regression was employed to identify the independent predictors of reproductive health service utilization among female adolescents. Accordingly, age of 16–20 years (AOR = 1.85, 95%CI: 1.17–2.92), occupation, educational level of their mothers (AOR = .33, 95%CI: .14–.77) and having a discussion with their family (AOR = 8.02, 9%CI: 5.52–11.66) were found to be significantly associated with reproductive health service utilization. Additionally, high perceived severity (AOR = 4.05, 95% CI: 2.68–6.11) and perceived barriers (AOR = .44, 95%CI: .30–64) were significant predictors of RHS utilization (Table 4).

**Table 4:** Multivariate logistic regression on reproductive health service utilization among female adolescents in Asgede-Tsimbla district, Northern Ethiopia, 2018

Variables	Utilization of reproductive health service		COR (95%, CI)	AOR [95%, CI]
	Non users [%] (*N* = 256)	Users *n* [%] (*N* = 588)		
Age				
1. 10–15	82 [43.6]	106 [56.4]	1	1
2. 16–20	230 [35.1]	426 [64.9]	*1.4 [1.03–1.99]*	***1.85 [1.17–2.92]***
Religion				
1. Orthodox Christian	275 [37.2]	464 [62.8]	1	1
2. Muslim	37 [35.2]	68 [64.8]	1.08 [.71–1.67]	.82 [.48–1.40]
Educational level				
1. Only read and write	27 [54.0]	23 [46.0]	0.4 [.22–74]	.78 [.37–1.62]
2. Grade 1–8	119 [37.8]	196 [62.2]	.79 [.58–1.08]	.96 [.65–1.41]
3. Grade 9–12	16 [28.6]	40 [71.4]	1.2 [.65–2.23]	***2.17 [1.04–4.50]***
4. College and above	138 [32.6]	285 [64.5]	1	1
Occupation				
1. Government Employee	20 [48.8]	21 [51.2]	1	1
2. Private Employee	34 [46.6]	39 [53.4]	1.09 [.50–2.35]	2.19 [.84–5.71]
3. Merchant	31 [34.8]	58 [65.2]	1.7 [.84–3.77]	***2.79 [1.11–6.96]***
4. Unemployed	47 [28.3]	119 [71.7]	2.4 [1.19–4.85]	***2.90 [1.19–7.06]***
5. Student	180 [37.9]	295 [62.2]	1.5 [.82–2.96]	***2.38 [1.04–5.42]***
Living Arrangement Or Live With:				
1. With Father and Mother	259 [35.6]	468 [64.4]	1	1
2. With Father or Mother only	31 [47.7]	34 [52.3]	.60 [.36–1.01]	.44 [.16–1.18]
3. With relatives/friends	5 [38.5]	8 [61.5]	.88 [.28–2.73]	.45 [.16–1.18]
4. Alone	17 [43.6]	22 [56.4]	.71 [.37–1.37]	.73 [.28–1.89]
Mother’s Educational Status				
1. Illiterate	83 [88.6]	38 [31.4]	.22 [.11–.46]	***.33 [.14–.77]***
2. Read and write	159 [30.9]	356 [69.1]	1.11 [.60–2.06]	.97 [.44–2.14]
3. Elementary school	34 [41.5]	48 [58.5]	.70 [.34–1.46]	.43 [.17–1.10]
4. Secondary School	19 [25.3]	56 [74.7]	1.47 [.67–3.21]	.89 [.36–2.19]
5. College and above	17 [33.3]	34 [66.7]	1	1
Marital Status of parents				
1. Live together	255 [36.2]	449 [63.8]	1	1
2. Divorced	23 [59.0]	16 [41.0]	.39 [.20–.76]	.35 [.14–.89]
3. Widowed	34 [33.7]	67 [66.3]	1.11 [.72–1.73]	1.86 [.76–4.52]
Discussion about reproductive health service with Family				
1. Yes	91 [35.5]	345 [58.7]	*2.00 [1.28–3.11]*	***8.02 [5.52–11.66]***
2. No	165 [64.5]	243 [41.3]	1	1
Knowledge^a^				
1. Less knowledgeable	268 [37.5]	447 [62.5]	1	1
2. Knowledgeable	32 [24.8]	97 [75.2]	1.81 [1.18–2.78]	1.61 [.98–2.67]
^b^Perceived Susceptibility^a^				
1. Low	26 [31.3]	57 [68.7]	1	1
2. High	274 [36.0]	487 [64.0]	1.1 [1.04–1.07]	1.84 [.95–3.55]
Perceived Severity^a^				
1. Low	104 [49.5]	106 [50.5]	1	1
2. High	196 [30.9]	438 [69.1]	4.2 [2.79–6.40]	***4.05 [2.68–6.11]***
Perceived Benefits^a^				
1. Low	48 [30.6]	109 [69.4]	1	1
2. High	252 [36.7]	435 [63.3]	0.76 [.52–1.10]	1.06 [.64–1.75]
Perceived Barriers^a^				
1. Low	94 [27.6]	246 [72.4]	1	1
2. High	206 [40.9.1]	296 [59.1]	1.80 [1.34–2.43]	***0.44[.33-64]***

Italics indicates statistically significant association at a *P* value < 0.05: ^a^Continuous variables; ^b^The reliability coefficient of the constructs of Health Belief Model for this study were 0.81, 0.78, 0.76 and 0.64 for perceived susceptibility, seriousness, barriers and benefits to utilization of reproductive health services respectively

## Discussion

This community-based study tried to assesses the reproductive health service utilization and its determinant among rural female adolescents. In this study, majority of the respondents (69.7%) utilized at least one type of reproductive health services. Among the reproductive health service users, 77.3% used family planning. This finding is similar to a study in that contraceptive use at first sexual intercourse was 64% [31]. This may imply that adolescents believe reproductive health service utilization could improve their life by decreasing reproductive health-related problems including some sexually related disease [32]. The current finding is however higher than the finding in South Ethiopia (47.2%) and 54.7% Shewa Ethiopia [33, 34]. This may be due to scocio-cultural differences and access to information.

In this study, the odds of using RHS were eight times more likely among female adolescents who used to discuss about RHS with their family. Supporting this finding, a study conducted in Amhara region indicated that parental monitoring was one of the significant predictors of RHS utilization among youths [34]. Conversely, female adolescents whose mothers are illiterate are less likely to utilize RHS implicating the knowledge of the mothers might affect the level of discussion or parental monitoring with regard to RHS utilization. Studies suggested that parents generally have talked to same-sex children about sexual issues. However, 87% of young women shared that they wanted more information from their fathers [35]. This study may justify that parent influence adolescents’ decisions around sex and reproductive matters. Besides, most girls think that the best way to learn about sex and health-related issues is through family [36, 37]. Other studies found that in both single and dual-parent households, mothers (74.2%) were much more likely to discuss about STDs such as HIV/AIDS than fathers (48.9%). In India, however, youth communication with parents on sensitive issues such as romantic relationships, reproduction, and contraception was rarely discussed and only reported by 2–6% of youths [38]. This inter-country and inter-regional difference may be directly related with social influence, cultural restrictions about reproductive health service.

As compared to the participants in the age range of 10–15 years, those in the age range of 16–20 years were 1.85 times more likely to utilize RHS which is consistent with the study conducted in south Ethiopia [33]. The odds of RHS utilization was also higher among Merchants (AOR = 2.78), unemployed (AOR = 2.9) and students (AOR = 2.38). This may be related to the relative maturity, sexual readiness or sexual experience and exposure to information in schools [39]. The other notable findings of this study indicated that female adolescents with high perceived severity towards reproductive health problems were four times more likely to use RHS. This study was in line with a study conducted on HIV in Addis Ababa, Ethiopia and Zambia in adults [40, 41]. The explanation towards this result may be high perceived severity towards reproductive health problems and its possible outcome may increase the perceived threat of an individual female adolescent who perceived as reproductive health problems are severe would be more likely to utilize the services. On the other hand, female adolescents with a high score of perceived barrier toward the utilization of reproductive health service were less likely to utilize the services. T When the barriers of health action outweigh the benefits in the minds of a given person the likelihood of taking action decreases [42]. The other implication of this study is that certain types of barriers are more or less important for particular cultures or norms. Modesty is a special barrier associated with lack of utilization among rural female adolescents. Similar explanation from the study in south-east Ethiopia, for unmarried adolescents, reproductive health services were offered as part of child health care and did not encompass sexual and reproductive health [43].

As a limitation, due to the sensitive nature of the information, accuracy of the information might be limited. Because the information was collected retrospectively, recall and social desirability biasmight have resulted in underreporting of the service utilized. Additionally, this study is focused on some components of adolescents reproductive health services (FP, health education, STI care, abortion care and VCT) while others components (such as antenatal care, delivery care, and post-natal care services, life skill education and condom use) are not addressed.

Ethiopia is working to create a generation free of reproductive health-related problems by the year 2030 including HIV/AIDS and other STIs. This demands strengthening reproductive health services utilization and related strategies which one of the curtail approaches may include addressing the reproductive health problems of adolescent females particularly who are living in the rural part of the country. Therefore, this finding will help to inform policymakers and healthcare planners working on the prevention of reproductive health problems at the local and national levels and makes a valuable contribution to the literature a front line study.

## Conclusions

Majority of the adolescent females were utilizing reproductive health services in the study area though it was very low when we compare it with the national plan. This study indicated that discussion on reproductive health matters with family, age, occupation, high score of perceived severity and perceived barriers were important predictors of reproductive health service utilization. Decreasing the perceived barriers to utilize reproductive health services would help further increase reproductive health services utilization among female adolescents; there should be community based health promotion activities in order to reach every segment of the adolescent population.

## Abbreviations

AIDS: Acquired immune deficiency syndrome
ASRH: Adolescent Sexual and Reproductive Health
DHS: Demographic and Health Survey
HBM: Health Believe Model
HEW: Health Extension Worker
HIV: Human Immunodeficiency Virus
IFH: Integrated Family Health
RH: Reproductive Health
RHP: Reproductive Health Package
STIs: Sexually Transmitted Infections
USA: United State of America
VCT: Voluntary Counseling Test
WHO: World Health Organization
